# L-Arginine Enhances Protein Synthesis by Phosphorylating mTOR (Thr 2446) in a Nitric Oxide-Dependent Manner in C2C12 Cells

**DOI:** 10.1155/2018/7569127

**Published:** 2018-04-26

**Authors:** Ruxia Wang, Hongchao Jiao, Jingpeng Zhao, Xiaojuan Wang, Hai Lin

**Affiliations:** Department of Animal Science, Shandong Key Lab for Animal Biotechnology and Disease Control, Shandong Agricultural University, Tai'an, Shandong, China

## Abstract

Muscle atrophy may arise from many factors such as inactivity, malnutrition, and inflammation. In the present study, we investigated the stimulatory effect of nitric oxide (NO) on muscle protein synthesis. Primarily, C2C12 cells were supplied with extra L-arginine (L-Arg) in the culture media. L-Arg supplementation increased the activity of inducible nitric oxide synthase (iNOS), the rate of protein synthesis, and the phosphorylation of mTOR (Thr 2446) and p70S6K (Thr 389). L-NAME, an NOS inhibitor, decreased NO concentrations within cells and abolished the stimulatory effect of L-Arg on protein synthesis and the phosphorylation of mTOR and p70S6K. In contrast, SNP (sodium nitroprusside), an NO donor, increased NO concentrations, enhanced protein synthesis, and upregulated mTOR and p70S6K phosphorylation, regardless of L-NAME treatment. Blocking mTOR with rapamycin abolished the stimulatory effect of both L-Arg and SNP on protein synthesis and p70S6K phosphorylation. These results indicate that L-Arg stimulates protein synthesis via the activation of the mTOR (Thr 2446)/p70S6K signaling pathway in an NO-dependent manner.

## 1. Introduction

Nitric oxide (NO) is a free radical that is produced by nitric oxide synthase (NOS) enzymes, which catalyze the conversion of L-arginine (L-Arg) to L-citrulline [1]. NO participates in specific signal transduction pathways, representing an important new paradigm in cell communication and signaling processes [2–4].

L-Arg, the precursor to NO, is involved in protein phosphorylation cascades and gene expression [5–7]. L-Arg supplementation enables burn patients to maintain muscle mass [8] and ameliorates muscle dysfunction in *mdx* mice (X-linked muscular dystrophy, a model of Duchenne muscular dystrophy) [9]. Additionally, the concentrations of specific free amino acids, notably arginine and glutamine, are associated with muscle growth and protein synthesis capacity during late pregnancy in well-nourished sheep [10, 11]. However, supplementation with citrulline, the metabolic precursor of arginine, did not result in therapeutically relevant outcomes such as increased skeletal muscle mass and peak muscle force in male mice suffering from 14 d of hindlimb immobilization [12]. Therefore, further study is required to determine whether L-Arg can stimulate protein synthesis in skeletal muscle tissue. Moreover, skeletal muscle participates in the overall NO metabolism by serving as a nitrate reservoir [13]. L-arginine could protect myocytes from wasting during catabolic conditions in an NO-independent manner [14]. L-citrulline, produced by arginine metabolism, protects skeletal muscle cells from cachectic stimuli in an iNOS-dependent manner [15]. NO is involved in the repair of skeletal muscle injury [16]. The activation of NO during muscle injury is critical in the early phases of the skeletal muscle repair process [17]. The maintenance of NO could ameliorate the symptoms of dystrophy [18, 19]. The age-related muscle refractoriness to exercise can be overcome with NO donor treatment [20]. There is growing evidence that NO is associated with skeletal muscle-wasting diseases, sarcopenia, and cachexia [21, 22]. However, the role of NO in myocyte protein synthesis under normal conditions also needs to be elucidated.

The mammalian target of rapamycin (mTOR), a serine/threonine protein kinase, plays a central role as a nutrient and energy sensor in skeletal muscle. The mTOR signaling pathway controls cell growth and metabolic progression by phosphorylating two downstream proteins: the eukaryotic initiation factor 4E-binding protein 1 (4E-BP1) and the ribosomal p70S6 kinase1 (p70S6K) [23, 24]. As a crucial component of the anabolic protein synthesis machinery, the mTOR pathway participates in the regulation of protein anabolism in skeletal muscle [25–27]. L-Arg protects muscle cells from wasting *in vitro* in an mTORC1-dependent manner [6, 14]. Therefore, we hypothesized that L-Arg is associated with the regulation of protein anabolism in myocytes through the involvement of the NO and mTOR/p70S6K signaling pathways.

In this study, the effect of L-Arg on myocyte protein synthesis and the involvement of NO were investigated *in vitro* in differentiated mouse C2C12 myoblasts. The protein synthesis rate was estimated with a nonradioactive method by labeling the newly synthesized polypeptides with low concentrations of puromycin and subsequently detecting these proteins with an anti-puromycin antibody [28, 29]. The involvement of the mTOR (Thr 2446)/p70S6K signaling pathway was also evaluated in this study.

## 2. Materials and Methods

### 2.1. Cell Culture

Mouse C2C12 myoblasts (China Center for Type Culture Collection, Wuhan, Hubei, CN) were plated and cultured in high-glucose Dulbecco's modified Eagle's medium (DMEM; Thermo Fisher, Shanghai, CN) with 10% fetal bovine serum (Gibco, Grand Island, NY, US) and 1% penicillin/streptomycin (Solarbio, Beijing, CN). At 80% confluence, the cells were induced to differentiate and form myotubes by culturing in DMEM supplemented with 2% horse serum (Gibco, Grand Island, NY, US) for 84 h. Before treatment, the medium was replaced with serum-free DMEM for 12 h. Finally, the C2C12 cells were exposed to the treatments detailed below. Each treatment was performed on 6 or 7 samples (*n* = 6 or 7).

### 2.2. L-Arginine, L-NAME, and SNP Treatments

The C2C12 cells were subjected to the following treatments: control (basal medium containing 398.7 *μ*M of L-Arg), extra L-arginine supplementation (1 mM; Sigma, Saint Louis, MO, US), *N*-nitro-L-arginine methyl ester (L-NAME; 10 mM; Sigma, Saint Louis, MO, US), and L-Arg supplementation (1 mM) plus L-NAME (10 mM). The treatment doses were selected based on previous studies [6, 14, 30]. To further evaluate the role of NO, C2C12 cultures were subjected to the following treatments: 1 *μ*M of sodium nitroprusside (SNP; an NO donor; Sigma, Saint Louis, MO, US) or SNP (1 *μ*M) plus L-NAME (10 mM). At 36 h after treatment, puromycin (10 *μ*M; Solarbio, Beijing, CN) was added to the culture media for an additional 30 min, and proteins were extracted from C2C12 cells and used for subsequent analyses.

### 2.3. Rapamycin Treatment

The C2C12 cells were treated with 100 nM of rapamycin (an inhibitor of p70S6K; Solarbio, Beijing, CN) for 30 min, followed by L-Arg (1 mM) or SNP (1 *μ*M) supplementation for 36 h. Thereafter, puromycin was added to the culture medium for an additional 30 min, and proteins were extracted from the C2C12 cells and used for subsequent analyses.

### 2.4. NO Concentration and NOS Activity Assays

NO concentrations in the media and cells were assessed using a commercial kit (Jiancheng Bioengineering Institute, Nanjing, Jiangsu, CN). NO is very chemically active and is thus easily converted into NO_2_^−^ and then NO_3_^−^. In this reaction system, the concentration of NO_2_^−^ was measured after conversion of NO_3_^−^ into NO_2_^−^ by nitrate reductase. The absorbance of the supernatant was determined at 550 nm using a spectrophotometer (Beijing PGeneral, Beijing, CN). Intracellular NOS activity, including total NOS enzymes (TNOS) and inducible NOS (iNOS), were determined using a commercial kit (Jiancheng Bioengineering Institute, Nanjing, Jiangsu, CN) according to the manufacturer's instructions. In the reaction system, NOS catalyzes L-arginine to produce NO, which reacts with nucleophilic substances to form nonferrous compounds. The absorbance was determined at 530 nm using a UV-2450 spectrophotometer (Beijing PGeneral, Beijing, CN). The experiment was also performed in the absence of calcium and the presence of a calcium chelator to determine the calcium-independent NOS activity, which was taken to represent iNOS activity.

### 2.5. Protein Preparation and Western Blotting

The cells were washed briefly with PBS (phosphate-buffered saline) and collected in 0.2 mL of RIPA (radio immunoprecipitation assay) lysis buffer (Beyotime, Haimen, Jiangsu, CN). Cell debris were removed by centrifugation at 12,000 ×g for 5 min at 4°C, and the supernatants were used for immunoblotting analysis. The BCA Protein Assay Kit (Beyotime, Nanjing, Jiangsu, CN) was used to determine protein concentrations. Aliquots containing 18 *μ*g of protein were separated by 7.5% SDS polyacrylamide gel electrophoresis, and the separated proteins were transferred onto polyvinylidene fluoride membranes (0.45 *μ*m; Millipore, Boston, MA, USA) at 200 mA for 2 h in western transfer buffer (Beyotime, Nanjing, Jiangsu, CN) containing 20% methanol. Membranes were then blocked for 1 h at room temperature and incubated at 4°C overnight with primary antibodies at an appropriate dilution ratio. The following primary antibodies were used: anti-phospho-4E-BP1 (Thr 37/46), anti-4E-BP1, anti-phospho-p70S6K (Thr 389), anti-p70S6K, anti-phospho-mTOR (Ser 2448), anti-mTOR (Cell Signaling Technologies, Danvers, MA, US), anti-phospho-mTOR (Ser 2481) and anti-phospho-mTOR (Thr 2446) (Abcam, Cambridge, MA, US), anti-mouse puromycin (Kerafast, Boston, MA, US), and anti-*β*-actin (Beyotime, Nanjing, Jiangsu, CN). After washing, the proteins were probed with horseradish peroxidase-linked anti-rabbit or anti-mouse secondary antibodies with gentle agitation for 4 h. The membranes were subsequently exposed to enhanced chemiluminescence plus western blot detection reagents (Beyotime, Nanjing, Jiangsu, CN). When two different proteins had the same or similar molecular weight, we used different membranes to separately detect them. In contrast, when two proteins were of different molecular weights or when the phosphorylated and total levels of one protein needed to be analyzed, the same membrane was used again. During this process, the membrane was blocked again and incubated with another antibody after one protein was detected. Finally, the membrane was scanned, and specific bands were quantified using Vilber Fusion FX7 Spectra (Vilber Lourmat, Paris, FR). The band intensity was normalized to the *β*-actin band in the same sample. For phosphorylated proteins, when the total protein bands showed significant differences in different treatments, the phosphorylated protein bands were normalized to the total protein bands. In contrast, if the total protein bands remained constant among different groups, both the phosphorylated and total protein bands were normalized to *β*-actin.

### 2.6. Protein Synthesis Rate Analysis

The protein synthesis rate was detected using a nonradioactive method [28]. The newly synthesized proteins labeled with puromycin were subsequently detected with an anti-puromycin antibody. The accumulation of puromycin-conjugated peptides into nascent peptide chains reflects the rate of protein synthesis [28, 29]. The protein-antibody complexes were detected with ECL Plus A and B (Beyotime, Nanjing, Jiangsu, CN), and the results were quantified using the Fusion FX software (Vilber Lourmat, Paris, FR).

### 2.7. Statistical Analysis

The data are expressed as the means ± SEM. The results were analyzed using one-way ANOVA and the Statistical Analysis Systems statistical software package (Version 8e; SAS Institute Inc., Cary, NC, US). For the observations of NO, TNOS, and iNOS at 3, 18, and 36 h time points, two-way ANOVA was used to estimate the main effects of L-Arg or L-NAME supplementation and time. Differences between the means were evaluated using Duncan's honestly significant difference tests. Differences were considered as significant at *P* < 0.05 and as approaching significance at *P* < 0.10.

## 3. Results

### 3.1. Effect of L-Arg on Protein Synthesis and mTOR and p70S6K Phosphorylation

**Figure 1:**
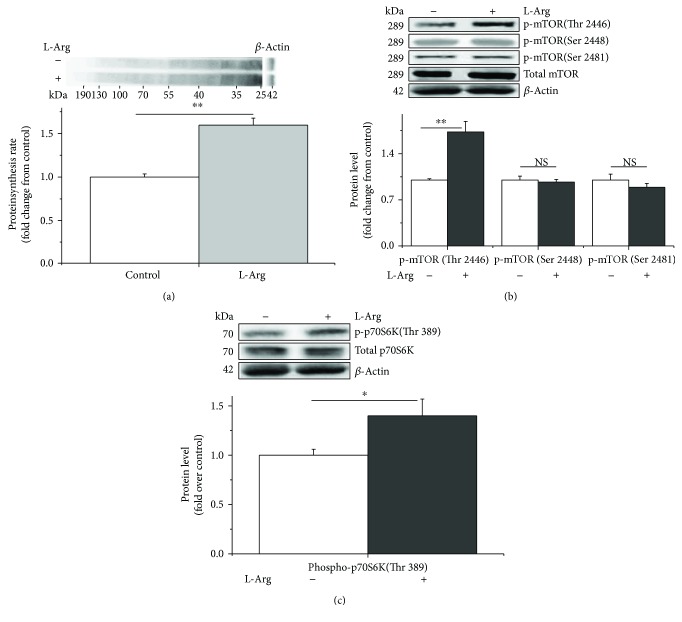
L-Arginine enhances protein synthesis by phosphorylating mTOR (Thr 2446) and p70S6K (Thr 389) in C2C12 cells. The protein synthesis rate was evaluated after treatment by supplementation with puromycin (10 *μ*M) for 30 min in the cell-free supernatant (a). The levels of phosphorylated mTOR (b) and p70S6K (c) in the C2C12 cells cultured for 36 h in the presence of 1 mM of L-arginine. When the total protein bands showed significant differences with different treatments, the phosphorylated protein bands were normalized to the total protein bands. In contrast, if the total protein bands were similar across different groups, both the phosphorylated and total protein bands were normalized to *β*-actin. Data are presented as the means ± SEM (*n* = 6); ^∗∗^*P* < 0.01 and ^∗^*P* < 0.05 compared with untreated cells. NS, *P* > 0.05.

**Figure 2:**
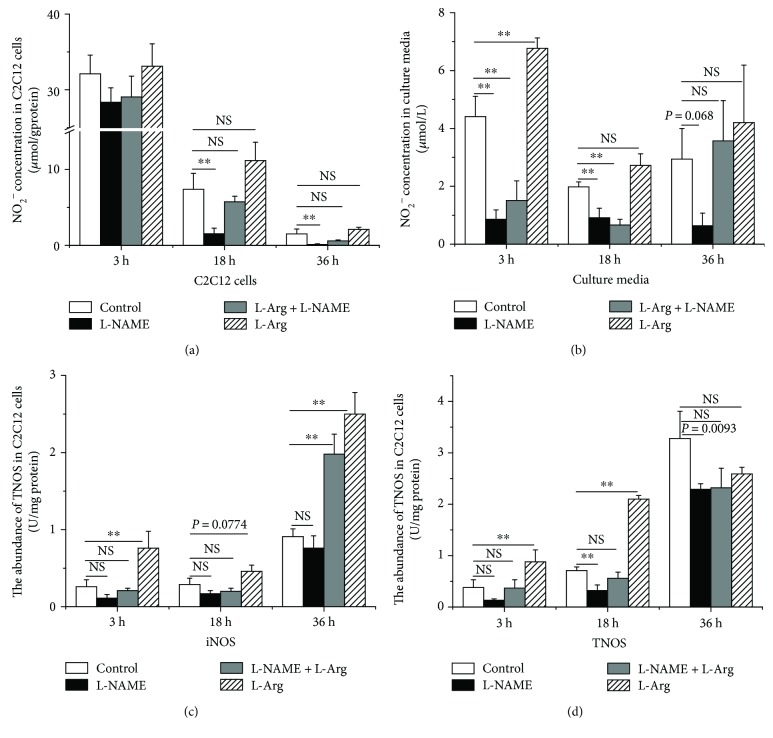
L-Arginine supplementation increases and L-NAME supplementation decreases NO concentrations in C2C12 cells and the culture medium, as well as the activities of inducible nitric oxide synthase (iNOS) and total nitric oxide synthase (TNOS) in C2C12 cells (U/mg protein). NO abundance in C2C12 cells (a) and culture medium (b) was detected after 3 h, 18 h, and 36 h treatments with L-arginine or L-NAME. The activity of NOS-iNOS (c) and TNOS (d) was analyzed following L-arginine or L-NAME supplementation for 3 h, 18 h, and 36 h in C2C12 cells. Data are expressed as the means ± SEM (*n* = 7). ^∗∗^*P* < 0.01 compared with untreated cells. NS, *P* > 0.05.

Compared with the control, L-Arg significantly increased protein synthesis (+70%, *P* < 0.05, Figure 1(a)). The levels of phospho-mTOR (Thr 2446) and phospho-p70S6K (Thr 389) were also significantly increased (+70% and +40%, *P* < 0.05, Figures 1(b) and 1(c), resp.). However, no differences (*P* > 0.05) were observed in the levels of phospho-mTOR (Ser 2448 and Ser 2481) (Figure 1(b)). Additionally, the NO abundance increased significantly in the L-Arg-supplemented culture medium at 3 h (+30%, *P* < 0.05, Figure 2(b)). The concentration of NO in the C2C12 cells and culture media tended to decrease with longer treatment times (−95% and −40%, *P* < 0.05, Figures 2(a) and 2(b), resp.). In contrast, the activities of iNOS and TNOS increased from 3 h to 36 h (+60% and +90%, *P* < 0.05, Figures 2(c) and 2(d), resp.). L-Arg significantly increased the activity of iNOS and TNOS at 3 h (+70% and +30%, *P* < 0.05, Figures 2(c) and 2(d), resp.). The activity of iNOS, however, was somewhat increased at 18 h (+35%, *P* = 0.0774, Figure 2(c)) and clearly increased at 36 h in the C2C12 cells (+65%, *P* < 0.05, Figure 2(c)).

### 3.2. Effect of L-NAME on Protein Synthesis and mTOR and p70S6K Phosphorylation

L-NAME decreased the NO abundance in the cell-free supernatants (−80%, Figure 2(b)) and NO levels in the C2C12 cells (−80% and −90%, *P* < 0.05) at 18 and 36 h (Figure 2(a)) and tended to decrease NO levels after 36 h treatment in the cell-free supernatants compared with those of the controls (−85%, *P* = 0.068, Figure 2(b)). iNOS activity was not significantly altered (*P* > 0.05) at 3 h, 18 h, and 36 h, whereas TNOS activity was significantly inhibited (−55%, *P* < 0.05) at 18 h (Figure 2(d)) and tended to be suppressed by L-NAME treatment in C2C12 cells at 36 h (−30%, *P* = 0.093) (Figure 2(d)). Compared with controls, however, L-Arg supplementation had no significant influence (*P* > 0.05) on NO concentrations in either the cells or the cell-free supernatant. Conversely, iNOS activity was significantly increased by L-Arg treatment (+60%, *P* < 0.05), regardless of the presence of L-NAME (Figure 2(c)) at 36 h. Additionally, TNOS activity was not altered by treatment with both L-Arg and L-NAME (*P* > 0.05) (Figure 2(d)).

**Figure 3:**
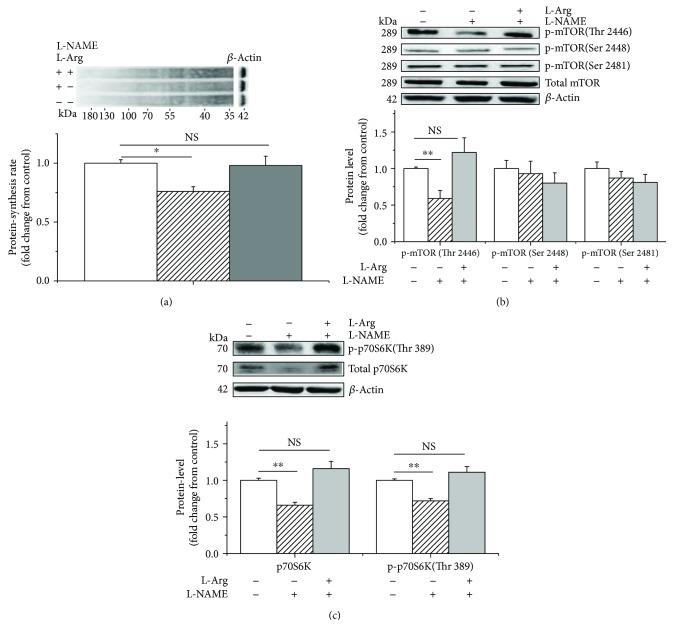
L-NAME and L-arginine treatment inhibits and evokes protein synthesis, respectively, in C2C12 cells. The protein synthesis rate was evaluated following treatment by supplementation with puromycin (10 *μ*M) for 30 min in the cell-free supernatant (a); the levels of phosphorylated mTOR (b) and p70S6K (c) following L-arginine (1 mM) supplementation in the presence of L-NAME (10 mM). When the total protein bands showed significant differences with different treatments, the phosphorylated protein bands were normalized to the total protein bands. In contrast, if the total protein bands were similar across different groups, both the phosphorylated and total protein bands were normalized to *β*-actin. Data are presented as the means ± SEM (*n* = 6). ^∗∗^*P* < 0.01 and ^∗^*P* < 0.05 compared with untreated cells. NS, *P* > 0.05.

L-NAME treatment significantly decreased (*P* < 0.05) protein synthesis (−25%, Figure 3(a)), as well as the phosphorylated mTOR (Thr 2446) (−40%, Figure 3(b)) and phospho-p70S6K (Thr 389) (−25%, Figure 3(c)) levels in the C2C12 cells. However, this inhibitory effect was significantly reduced by supplementation with 1 mM of L-Arg (+25%, +50%, and +35%, *P* < 0.05, Figures 3(a)–3(c)). In contrast, no difference was detected (*P* > 0.05) in the levels of the total or phosphorylated mTOR (Ser 2448 and Ser 2481) (Figure 3(b)).

### 3.3. Effect of SNP on Protein Synthesis and mTOR, p70S6K, and 4E-BP1 Phosphorylation

**Figure 4:**
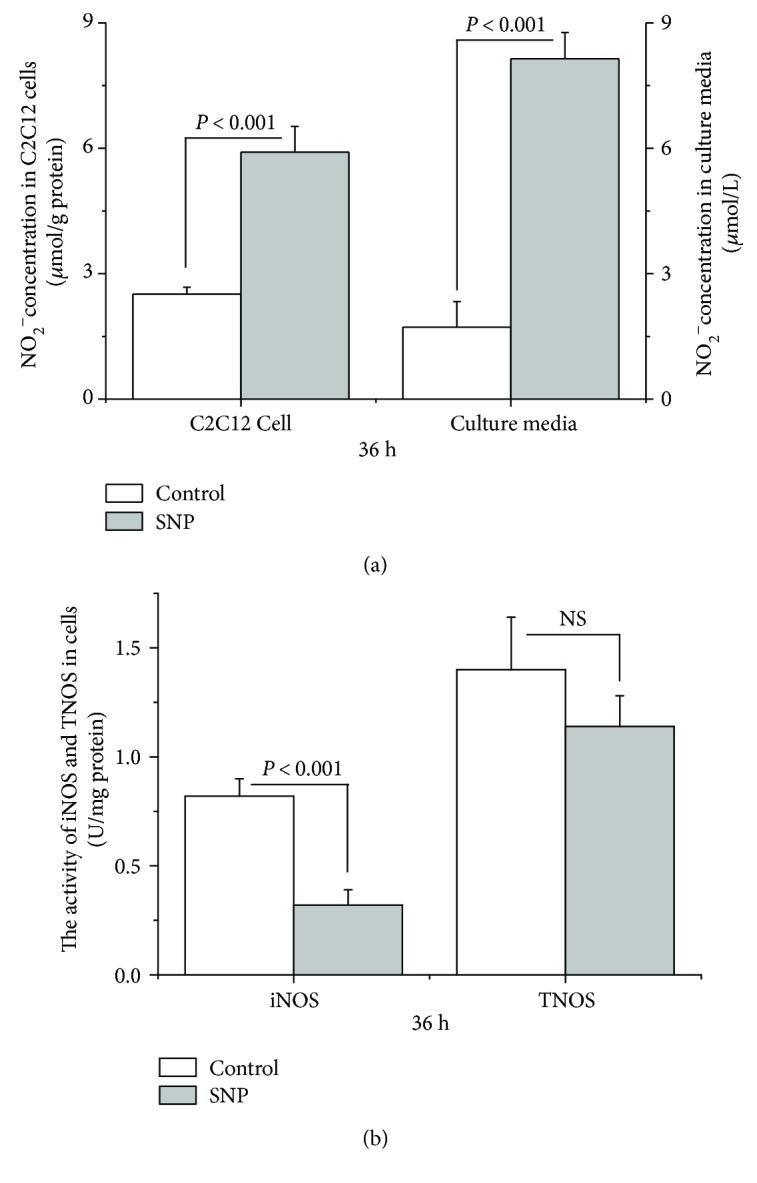
SNP supplementation increases NO concentration in C2C12 cells and the culture medium and inhibits the activity of inducible nitric oxide synthase (iNOS) in C2C12 cells (U/mg protein). Data are expressed as the means ± SEM (*n* = 7). NS, *P* > 0.05.

To further evaluate whether NO is involved in the regulation of protein synthesis, we tested the effect of SNP, an NO donor. The results showed that SNP treatment increased NO concentrations in the C2C12 cells (+55%, *P* < 0.05) and in the cell-free supernatants (+80%, *P* < 0.05, Figure 4(a)). Furthermore, iNOS (−60%, *P* < 0.05), but not TNOS (*P* > 0.05), activity was suppressed by SNP treatment (Figure 4(b)).

**Figure 5:**
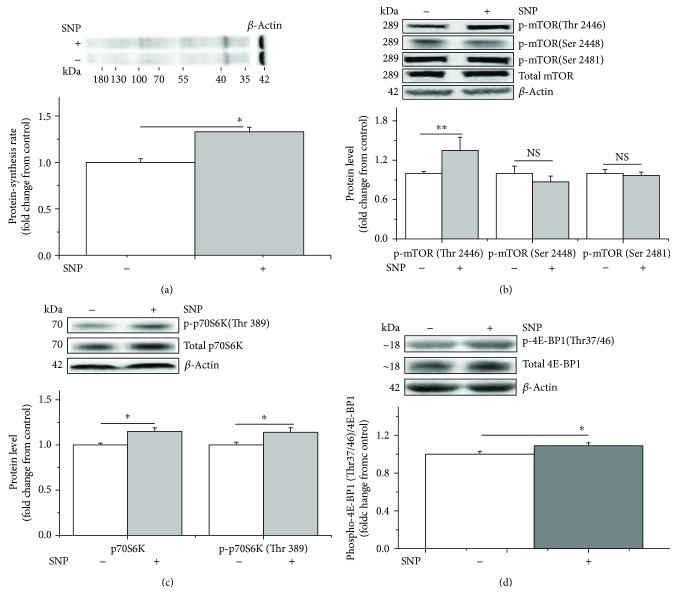
SNP treatment increases the protein synthesis rate and the phosphorylated levels of mTOR (Thr 2446), p70S6K (Thr 389), and 4E-BP1 (Thr 37/46) in C2C12 cells. The protein synthesis rate was evaluated following treatment by supplementation with puromycin (10 *μ*M) for 30 min in the cell-free supernatant (a). Levels of phosphorylated mTOR (b), p70S6K (c), and 4E-BP1 (d) in C2C12 cells in the presence of 1 *μ*M of SNP. When the total protein bands showed significant differences with different treatments, the phosphorylated protein bands were normalized to the total protein bands. In contrast, if the total protein bands were similar across different groups, both the phosphorylated and total protein bands were normalized to *β*-actin. Data are presented as the means ± SEM (*n* = 6). ^∗∗^*P* < 0.01 and ^∗^*P* < 0.05 compared with untreated cells. NS, *P* > 0.05.

The results also indicated that SNP significantly increased protein synthesis (+30%, *P* < 0.05, Figure 5(a)), increased the phosphorylation of mTOR (Thr 2446) (35%, *P* < 0.05, Figure 5(b)), and upregulated both total p70S6K and phospho-p70S6K (Thr 389) (+15% and +15%, *P* < 0.05, Figure 5(c)) and phosphor-4E-BP1 (Thr 37/46) levels (+10%, *P* < 0.05, Figure 5(d)). In contrast, phospho-mTOR (Ser 2448 and Ser 2481) levels remained unaltered (*P* > 0.05, Figure 5(d)).

**Figure 6:**
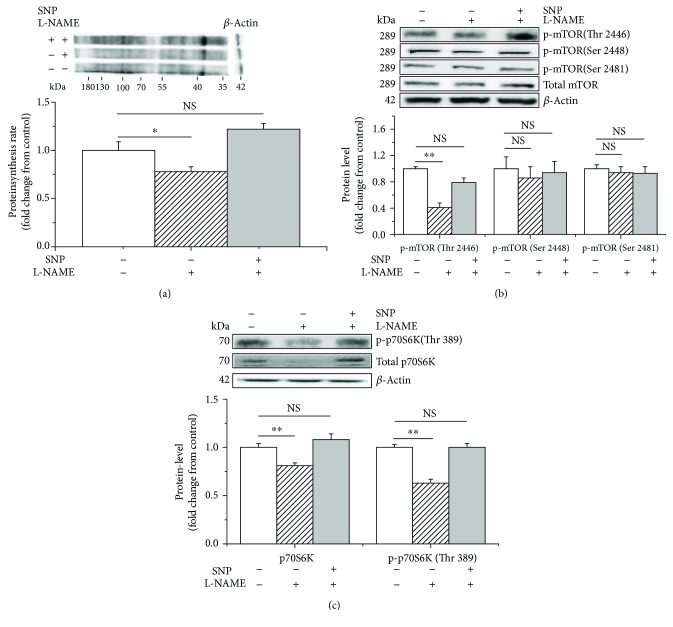
L-NAME and SNP, respectively, inhibit and activate protein synthesis and the phosphorylation of mTOR (Thr 2446) and p70S6K (Thr 389) in C2C12 cells. The protein synthesis rate was evaluated following treatment by supplementation with puromycin (10 *μ*M) for 30 min in the cell-free supernatant (a). The phosphorylation levels of mTOR (b) and p70S6K (c) in C2C12 cells cultured for 36 h in the presence of 1 *μ*M of SNP and 10 mM of L-NAME. When the total protein bands showed significant differences with different treatments, the phosphorylated protein bands were normalized to the total protein bands. In contrast, if the total protein bands were similar across different groups, both the phosphorylated and total protein bands were normalized to *β*-actin. Data are presented as the means ± SEM (*n* = 6). ^∗∗^*P* < 0.01 and ^∗^*P* < 0.05 compared with untreated cells. NS, *P* > 0.05.

The effect of SNP on L-NAME-induced suppression of protein synthesis was further investigated. L-NAME significantly inhibited the protein synthesis rate (−20%, *P* < 0.05, Figure 6(a)), downregulated the levels of phosphorylated mTOR (Thr 2446) (−60%, *P* < 0.05, Figure 6(b)), and decreased the phosphorylation of p70S6K (Thr 389) (−35%, *P* < 0.05, Figure 6(c)). In contrast, SNP supplementation significantly alleviated the L-NAME-induced inhibition of protein synthesis (+40%, *P* < 0.05, Figure 6(a)). SNP supplementation also upregulated the phosphorylated mTOR (Thr 2446), as well as the total and phosphorylated p70S6K (Thr 389), levels compared with those of the L-NAME treatment (+40% and +35%, *P* < 0.05, Figures 6(b) and 6(c)). However, no difference was detected between the SNP and control treatments (*P* > 0.05). Neither SNP nor L-NAME treatment altered the levels of the phosphorylated mTOR (Ser 2448 and Ser 2481) (*P* > 0.05, Figure 6(b)).

### 3.4. Effect of Rapamycin Treatment on Protein Synthesis and mTOR and p70S6K Phosphorylation

**Figure 7:**
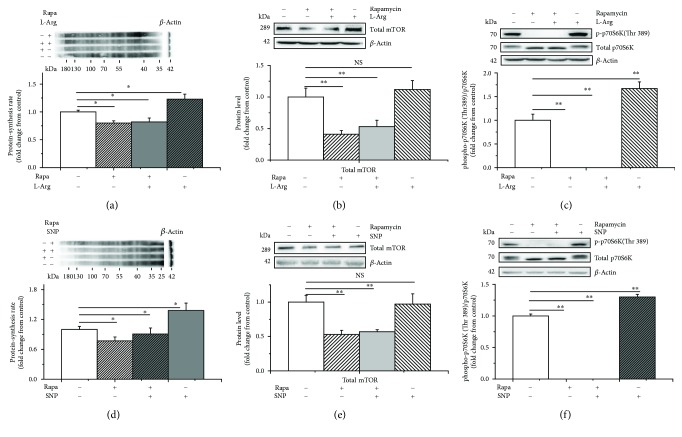
Rapamycin inhibits protein synthesis as well as mTOR and p70S6K in C2C12 cells. The protein synthesis rate was evaluated following treatment by supplementation with puromycin (10 *μ*M) for 30 min in the cell-free supernatant (a, d); the level of total mTOR (b, e) and p70S6K (c, f) following treatment with 1 mM of L-arginine or 1 *μ*M of SNP in the presence of rapamycin (100 nM). When the total protein bands showed significant differences with different treatments, the phosphorylated protein bands were normalized to the total protein bands. In contrast, if the total protein bands were similar across different groups, both the phosphorylated and total protein bands were normalized to *β*-actin. Data are presented as the means ± SEM (*n* = 6). ^∗∗^*P* < 0.01 and ^∗^*P* < 0.05 compared with untreated cells. NS, *P* > 0.05.

We further verified whether p70S6K is involved in the stimulatory effect of NO on protein synthesis by blocking mTOR/p70S6K signaling. The results showed that the protein synthesis rate significantly decreased with rapamycin treatment (−20% and −25%, *P* < 0.05, Figures 7(a) and 7(d)). Meanwhile, rapamycin treatment decreased the levels of total mTOR (−60% and −50%, *P* < 0.05, Figures 7(b) and 7(e)) and p70S6K (Thr 389) phosphorylation (−100% and −100%, *P* < 0.05, Figures 7(c) and 7(f)). Moreover, supplementation with L-Arg or SNP did not reverse the effects of rapamycin treatment on the C2C12 cells (*P* > 0.05) (Figures 7(a) and 7(e)).

## 4. Discussion

In the present study, we investigated the role of L-Arg on *in vitro* muscle protein synthesis under normal conditions. The results indicated that L-Arg supplementation stimulated protein synthesis, increased p70S6K phosphorylation (Thr 389), and upregulated phosphorylated mTOR (Thr 2446) levels. The stimulatory effect of L-Arg on protein synthesis and p70S6K (Thr 389) and mTOR (Thr 2446) phosphorylation was abolished by the presence of L-NAME, an NOS inhibitor. In contrast, SNP, an NO donor, increased protein synthesis and upregulated both p70S6K and mTOR phosphorylation (Thr 389 and Thr 2446, resp.); this effect was not altered by L-NAME. Blocking the phosphorylation of p70S6K with rapamycin, however, abolished the stimulatory effect of both L-Arg and SNP on protein synthesis. These results demonstrate that NO is associated with the regulation of muscle protein synthesis via the mTOR/p70S6K pathway. Moreover, except for one mTOR phosphorylation site (Thr 2446), the phosphorylated levels of mTOR (Ser 2448 and Ser 2481) were not altered by the L-Arg, L-NAME, or SNP treatment, suggesting that this specific mTOR site (Thr 2446) is the target site involved in the regulation of protein synthesis by NO.

### 4.1. L-Arg Enhanced Protein Synthesis and the Phosphorylation of p70S6K (Thr 389) and mTOR (Thr 2446)

In animal experiments, L-Arg has been demonstrated to enhance protein synthesis in skeletal muscle [31, 32]. Moreover, L-Arg supplementation is beneficial for the maintenance of muscle mass in burn patients and ameliorates the muscle dysfunction associated with *mdx* mice [8, 9]. The advantageous effect of L-Arg could be partially accounted for by the increased blood circulation in skeletal muscles [33–35].

In the present study, we further investigated the direct role of L-Arg in muscle cell protein synthesis. We confirmed the enhancement of protein synthesis in C2C12 cells following L-Arg supplementation, which was in line with the results of [35], in which arginine was found to protect myocytes from wasting by stimulating protein synthesis during catabolic conditions in C2C12 cells. These results suggest that L-Arg stimulates protein synthesis in muscle cells regardless of nutritional status; this finding is supported by the observation of Sales et al. [10, 11], who reported that free amino acids, especially arginine, within muscle cells may be associated with protein synthesis capacity in fetal lambs of well-nourished sheep.

mTOR complex 1 (mTORC1), consisting of Raptor, G*β*L, PRAS40, and DEPTOR, phosphorylates 4E-BP1 and p70S6K and thus stimulates protein synthesis [36]. *In vivo*, L-Arg treatment was shown to stimulate p70S6K phosphorylation [32]. Under wasting conditions, arginine treatment increased the levels of phosphorylated mTOR, p70S6K, and 4E-BP1 in C2C12 cells [14]. In line with previous studies, the present results indicated that L-Arg supplementation upregulated p70S6K (Thr 389) and mTOR (Thr 2446) phosphorylation levels, which suggested that the mTOR/p70S6K signaling pathway is an intracellular target of L-Arg. In contrast to the work by Ham et al. [14], who reported that arginine evoked mTOR phosphorylation (Ser 2448) in C2C12 cells under wasting conditions, we observed that L-Arg stimulated the phosphorylation of mTOR at Thr2446, but not at Ser2448 or Ser2481, in C2C12 cells under normal nutritional conditions, suggesting that L-Arg may modulate mTOR activity at different sites according to cellular nutritional state.

### 4.2. L-Arg-Stimulated Protein Synthesis via NO

In humans, skeletal muscle participates in the overall NO metabolism by serving as a nitrate reservoir [13]. As the precursor of NO, L-Arg has been shown to be involved in protein phosphorylation cascades and gene expression by serving as a cell signaling molecule [5, 37]. Arginine, a conditionally essential amino acid, is known to participate in the production of NO [38]. Under catabolic conditions, L-Arg was also found to exhibit NO-independent protective effects on muscle wasting [14]. To further investigate the role of NO in the regulation of arginine, we first used L-NAME to suppress NOS activity. The significant decrease in cellular NO concentrations and the suppression of TNOS activity indicated that L-NAME decreased NO production. The decreased protein synthesis and levels of phosphorylated p70S6K (Thr 389) and mTOR (Thr 2446) caused by L-NAME treatment suggested that NO may be involved in muscle cell protein synthesis via the mTOR/p70S6K pathway. The increased NO concentrations in the cell-free supernatant and the restoration of iNOS activity upon treatment with both L-NAME and L-Arg indicated that the suppressive effect of L-NAME on NOS activity could be relieved by L-Arg supplementation. Consistent with this result, L-Arg supplementation reversed the effects of L-NAME on protein synthesis and p70S6K phosphorylation, suggesting that either NO or arginine is involved in the modulation of protein synthesis.

To further verify this hypothesis, we evaluated the effect of SNP, an NO donor, on protein synthesis and p70S6K and mTOR phosphorylation. The elevated NO production in cells and in the media, as well as the decreased iNOS activity, indicated that SNP treatment provides sufficient NO independent of NOS. The increased protein synthesis caused by SNP also suggests that NO, rather than L-Arg, is associated with the regulation of protein synthesis in muscle cells. This hypothesis was confirmed by the observation that the negative effect of L-NAME on protein synthesis could be rescued by SNP supplementation. Therefore, the present results demonstrate that NO, rather than L-Arg, was associated with the regulation of protein synthesis in C2C12 cells, which is consistent with the mechanism in intestinal epithelial cells [39]. This result was in line with previous work showing that the maintenance of NO could ameliorate dystrophy symptoms [18, 19]. The age-related muscle refractoriness to exercise can be overcome with NO donor treatment [20]. This result, however, contradicted the work of Ham et al., who reported that L-arginine reduces muscle wasting in a dose-dependent manner through NO-independent activation of mTOR [14]. The nutritional status of the cells may account for these different observations. In the present study, we investigated the effect of arginine and NO on protein synthesis and the activation of the mTOR/p70S6K signaling pathway in cells under normal, but not nutrient-deprived, conditions. These results may imply that L-Arg and NO have a positive effect on muscle protein synthesis in a nutritional status-dependent manner.

The SNP-induced upregulation of p70S6K and 4E-BP1 phosphorylation suggests that p70S6K and 4E-BP1 are the target proteins of NO, which is consistent with previous studies [40, 41]. However, SNP supplementation only partially restored the downregulation of p70S6K (Thr 389) and mTOR (Thr 2446) phosphorylation by L-NAME. To further clarify the effect of NO on mTOR and p70S6K, the C2C12 cells were treated with rapamycin. The suppression of protein synthesis and inhibition of p70S6K and mTOR phosphorylation by rapamycin indicated that the mTOR/p70S6K pathway is an important pathway in muscle cell protein synthesis, which is in line with previous studies [42, 43]. The suppressive effect of rapamycin was not reversed by either L-Arg or SNP, which suggests that the regulatory effect of L-Arg or NO on muscle cell protein synthesis is dependent on the phosphorylation of mTOR specifically at Thr 2446 (rather than at Ser 2448 or Ser 2481) to initiate the phosphorylation of p70S6K (Thr 389) and 4E-BP1 (Thr 37/46). Further, the L-Arg- or SNP-induced activation of mTOR (Thr 2446), p70S6K (Thr 389), and 4E-BP1 (Thr 37/46) is consistent with recent research in cocaine treatment [44]. Even though the C-terminus of mTOR contains the phosphorylation sites Thr 2446, Ser 2448, and Ser 2481, which lie within or near a repressor domain and consequently correlate with an increase in activity [45], these sites are regulated by several different kinases including downstream effectors of the mTOR pathway itself or by autophosphorylation. For example, Thr 2446 is a target of AMP-activated protein kinase (AMPK) and S6K [46, 47], which is a novel mammalian target of the rapamycin (mTOR) phosphorylation site regulated by nutrient status [46] and is involved in various metabolic processes. Ser 2481 is an autophosphorylation site that directly monitors the catalytic activity of both mTORC1 and mTORC2 [48, 49]. The Ser 2448 site is also a key mTOR phosphorylation site and is regulated by Akt and S6K [50, 51]. We made the interesting observation that the phosphorylation status of the Ser 2481 and Ser 2448 sites did not change following L-arginine treatment or SNP supplementation. The specific L-arginine- and SNP-induced mTOR phosphorylation pattern is indicative of upstream signaling, as each phosphorylation site is regulated by different mechanisms.

NO participates in cellular signal transduction mainly through S-nitrosylation of allosteric and active-site cysteine thiols within proteins [37, 52, 53]. In aged rats (33 months), the increased phosphorylation of Akt (Ser 473 and Thr 308) in soleus muscles is associated with diminished mTOR phosphorylation, whereas the age-related impairment in Akt kinase activity is associated with increases in Akt S-nitrosylation [54]. Hence, the actions of NO in skeletal muscle under different physiological conditions need to be further investigated.

### 4.3. The Role of NOS on Muscle Protein Synthesis

NOS has three isoforms: iNOS, nNOS (neuronal NOS, type I), and eNOS (endothelial NOS, type II). In skeletal muscle, nNOS is the major NOS isoform. There is growing evidence that NOS is associated with the development of muscle atrophy. The nNOS/NO system modulates muscle functions such as insulin sensitivity and glucose uptake, muscle contraction, vasodilation, and activation of satellite cells [37, 55, 56]. The translocation of nNOS from the sarcolemma to the cytoplasm, however, is involved in muscle atrophy in an uploading model mimicked by tail suspension [57, 58] and in prolonged alcoholic myopathy [59]. NO signaling is dysregulated during muscular dystrophy due to the disruption of the dystrophin glycoprotein complex (DGC), which anchors nNOS [60]. The inhibition of tendon NOS contributes to the attenuation of atrophy and acceleration of muscle regeneration [61]. On the other hand, iNOS is expressed exclusively in the presence of proinflammatory cytokines. iNOS has been proven to be an important mediator in TNF*α*-induced cachectic muscle loss and in age-related muscle wasting (sarcopenia) [21]. Under pathological conditions, the activation of iNOS promotes muscle atrophy [62]. NO may exert both protective and pathological effects during muscle wasting, depending on quantitative effects as well as on the spatial arrangement of NOS [22]. L-Citrulline preserves protein synthesis rates and protects myotubes from wasting through induction of the iNOS isoform [15]. In the present study, protein synthesis was suppressed by L-NAME treatment and stimulated by L-Arg. L-NAME suppressed TNOS activity at 18 h (*P* < 0.01) and 36 h (*P* = 0.093) but had little influence on iNOS, suggesting that TNOS may be responsible for blocking protein synthesis. In contrast, L-Arg supplementation increased iNOS and TNOS at different time points. Hence, the role of iNOS and nNOS on protein synthesis and the mTOR/p70S6K pathway requires further study.

## 5. Conclusion

**Figure 8:**
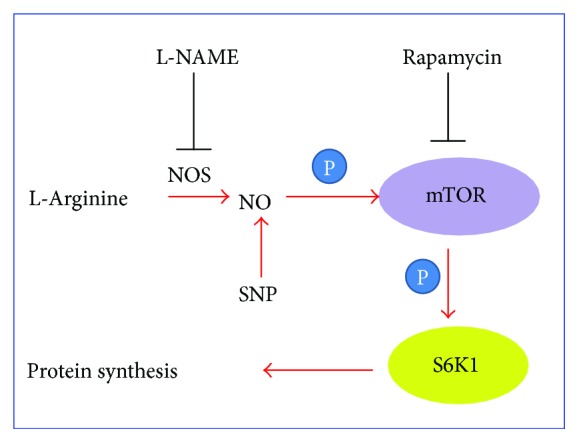
The working model of L-arginine/NO in muscle protein synthesis. L-NAME: *N*-nitro-L-arginine methyl ester; NO: nitric oxide; NOS: nitric oxide synthase; SNP: sodium nitroprusside.

In conclusion, our results demonstrate that L-Arg is associated with the regulation of muscle development via the mTOR (Thr 2446)/p70S6K signaling pathway in an NO-dependent manner (Figure 8). These results highlight the potential clinical application of L-Arg or NO for the modulation of muscle metabolism.
